# Leveraging the sugarcane CRISPR/Cas9 technique for genetic improvement of non-cultivated grasses

**DOI:** 10.3389/fpls.2024.1369416

**Published:** 2024-03-27

**Authors:** Chunjia Li, Muhammad Aamir Iqbal

**Affiliations:** ^1^ National Key Laboratory for Biological Breeding of Tropical Crops, Kunming, Yunnan, China; ^2^ Sugarcane Research Institute, Yunnan Academy of Agricultural Sciences/Yunnan Key Laboratory of Sugarcane Genetic Improvement, Kaiyuan, Yunnan, China

**Keywords:** agrobacterium, genetic transformation, polyploidy, steppe and pampas, transgenic plants, lemon grass, wild sugarcane

## Abstract

Under changing climatic scenarios, grassland conservation and development have become imperative to impart functional sustainability to their ecosystem services. These goals could be effectively and efficiently achieved with targeted genetic improvement of native grass species. To the best of our literature search, very scant research findings are available pertaining to gene editing of non-cultivated grass species (switch grass, wild sugarcane, Prairie cordgrass, Bermuda grass, Chinese silver grass, etc.) prevalent in natural and semi-natural grasslands. Thus, to explore this novel research aspect, this study purposes that gene editing techniques employed for improvement of cultivated grasses especially sugarcane might be used for non-cultivated grasses as well. Our hypothesis behind suggesting sugarcane as a model crop for genetic improvement of non-cultivated grasses is the intricacy of gene editing owing to polyploidy and aneuploidy compared to other cultivated grasses (rice, wheat, barley, maize, etc.). Another reason is that genome editing protocols in sugarcane (*x* = 10–13) have been developed and optimized, taking into consideration the high level of genetic redundancy. Thus, as per our knowledge, this review is the first study that objectively evaluates the concept and functioning of the CRISPR (clustered regularly interspaced short palindromic repeats)/Cas9 technique in sugarcane regarding high versatility, target specificity, efficiency, design simplicity, and multiplexing capacity in order to explore novel research perspectives for gene editing of non-cultivated grasses against biotic and abiotic stresses. Additionally, pronounced challenges confronting sugarcane gene editing have resulted in the development of different variants (Cas9, Cas12a, Cas12b, and SpRY) of the CRISPR tool, whose technicalities have also been critically assessed. Moreover, different limitations of this technique that could emerge during gene editing of non-cultivated grass species have also been highlighted.

## 1 Introduction

**Figure 1:**
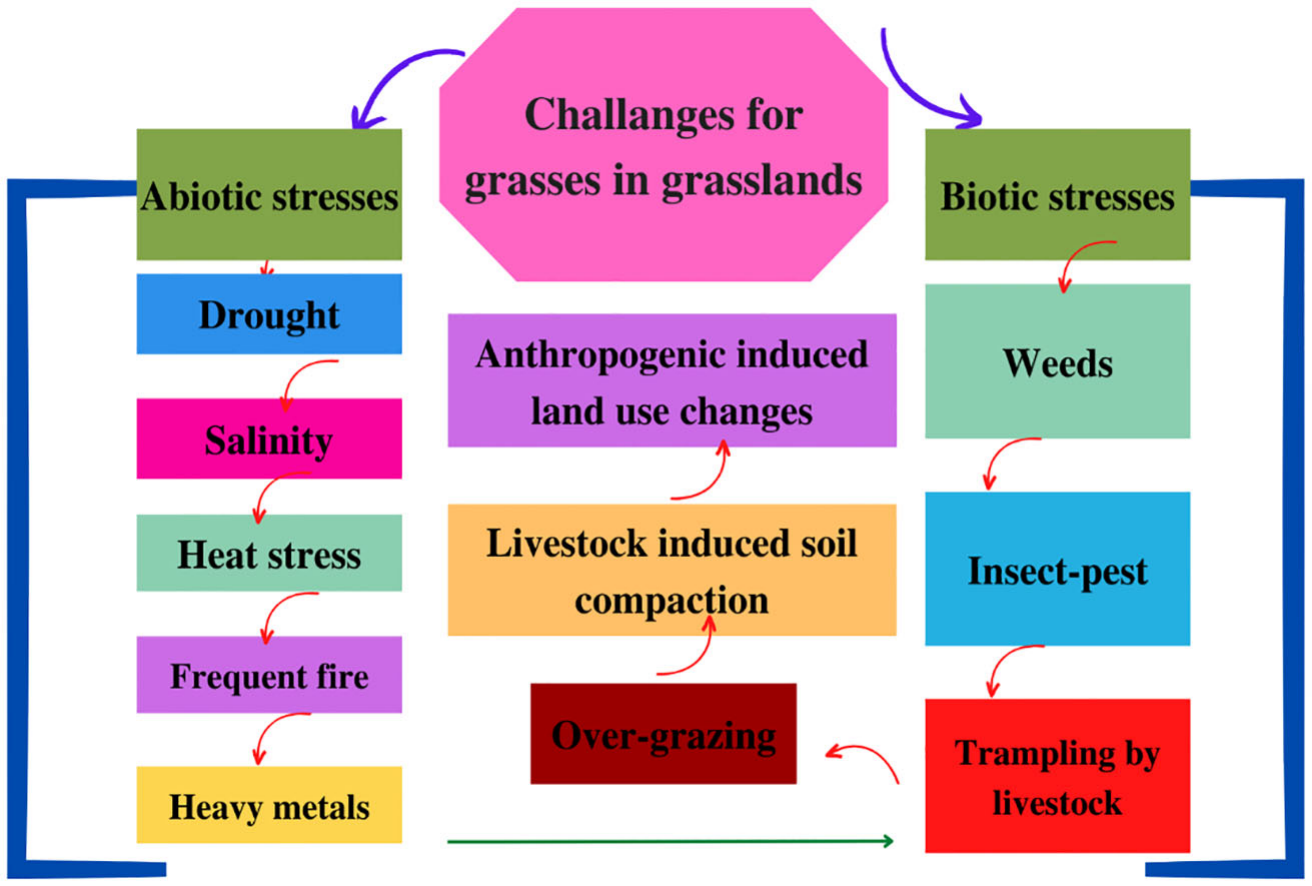
Pronounced abiotic and biotic stresses affect grasses in grasslands, reducing their ecosystem services and necessitating genome editing of grass species for imparting tolerance against biotic and abiotic stresses along with improving their productivity.

Globally, grasslands are considered the biggest ecosystem and serve as a carbon sink, ecological barriers, a watershed for low riparian regions, feedstock for ruminants, and mineral extraction sites for drilling and mining, and offer numerous associated benefits like wool, herbs for traditional medicines, tourism, and leisure (Wen et al., 2018; Iqbal, 2022). Recently, it has become imperative to conserve grasslands by employing practices that ensure protection and sustainable management of grassland ecosystems by maintaining the biodiversity and ecological integrity for persistent provision of ecosystem services (Abbas et al., 2015; Yu et al., 2019). Contrastingly, different initiatives intended for improving the productivity and sustainability of grasslands for agricultural purposes have been termed as grassland development (Yang et al., 2016; Iqbal et al., 2022; Ijaz et al., 2023). However, grassland conservation and development have remained neglected owing to a multitude of challenges especially climate change (CC). Additionally, overgrazing by livestock has caused serious depletion of grass resources along with adversely affecting the sustainability and health of natural and semi-natural grasslands (Wu et al., 2009; Wen et al., 2018). In addition, soil erosion (a soil quality degradation process that negatively impacts the health of grasses and the entire ecosystem), biodiversity loss, and, more importantly, the invasion of noxious weeds have reduced the productivity of native grass species. The invasive plant species tend to outcompete native grasses, which ultimately alters the grassland’s ecosystem composition and balance (Su et al., 2015; Wen et al., 2018). The underlying reason is that invasive plant species having aggressive growth patterns tend to acquire more growth resources and ultimately disrupt the ecosystem balance by overcoming native grass species (Maqsood et al., 2020; Abbas et al., 2021). Recently, the need for agricultural expansion owing to increasing food demand, rapid urbanization, and numerous abrupt land-use changes has caused grassland conversion into croplands primarily owing to the low productivity of grasses (Liu et al., 2008; Yu et al., 2019). More importantly, global CC has seriously affected grasslands owing to altering temperature and precipitation patterns. Likewise, persistent CC causes periodic fires (planned as well as wild) that are traditionally believed to stimulate the growth of grasses along with controlling the woody vegetation (Abbas et al., 2015). However, fire mismanagement leads to woody plants’ encroachment, which ultimately reduces suitable habitat availability for grass species (Sun et al., 2013; Wang et al., 2016; Yu et al., 2019). Recently, native grasses are exposed to water scarcity owing to changes in precipitation patterns along with other stresses including heat, salinity, and pollution. **Figure 1** illustrates the pronounced challenges (environmental, ecological, and anthropogenic) faced by grass species in grasslands. The net result of all these stresses is a significant loss of habitat, which has threatened the survival of grass species; all these stresses have led to a serious decline in ecosystem services provided by grasslands (Di et al., 2014; Dong et al., 2019; Iqbal et al., 2022). Therefore, grassland conservation and development are directly linked to genetic improvement of grasses as CC has posed varying challenges to native grass species. Moreover, different anthropogenic, environmental, ecological, and soil-related challenges are faced by grasses in natural or improved grasslands, which necessitate their genetic improvement in order to impart sustainability to grassland ecosystems.

The non-cultivated grasses’ genetic improvement has remained neglected owing to the focus on major cultivated grasses like wheat, rice, maize, and sugarcane. In addition, most non-cultivated grasses are polyploidy, which restricts gene editing using traditional approaches. Modern gene editing techniques especially CRISPR (clustered regularly interspaced short palindromic repeats) have effectively inserted and knocked out targeted genes in cultivated grasses for boosting yield attributes under environmental extremes (Xing et al., 2014; Svitashev et al., 2015; Waltz, 2016; Gasparis et al., 2018; Holubová et al., 2018; Brauer et al., 2020). Moreover, this technique has been employed to study gene functions through selective disruption of genes and thereafter observing the resulting effects of altered genes in cultivated grasses (Kim et al., 2022). However, the genome editing of non-cultivated grasses might be initiated by taking sugarcane as a model plant because it is a perennial C-4 grass having exceptional potential for converting solar radiation and farm inputs (nutrients, water, etc.) into chemical harvestable energy (sucrose) (Hoang et al., 2015; Hussin et al., 2022). Furthermore, sugarcane has demonstrated intricacy in its genome editing owing to polyploidy and aneuploidy (Eid et al., 2021). Despite these challenges, genome editing techniques employed in sugarcane have improved yield attributes and plant metabolism, leading to enhanced yield on a sustainable basis (Jung and Altpeter, 2016). More importantly, numerous genetic modifications have been introduced for conferring resistance against diseases that adversely affect sugarcane growth, yield, and sucrose recovery (Viswanathan and Rao, 2011; Ali et al., 2019; Afzal et al., 2020; Wu et al., 2023). Targeted gene editing has assisted in producing sugarcane varieties that are resistant against pests and thus need fewer chemical pesticides. Moreover, these newly developed varieties have the potential to tolerate abiotic stresses (drought, heat, salinity, water logging, etc.) (Ramiro et al., 2016; Hussain et al., 2018; Li et al., 2018; Budeguer et al., 2021).

Thus, to the best of our understanding, this synthesis review is the first study that describes sugarcane as a model crop (because it is a perennial grass and presents high intricacy of gene editing owing to polyploidy), suggesting genetic improvement in non-cultivated grass species. Another reason is genome editing protocols of sugarcane hold bright perspectives for non-cultivated grass improvement because gene editing techniques in sugarcane (*x* = 10–13) have been developed and subsequently optimized considering the high level of genetic redundancy. Among gene editing techniques, special emphasis has been placed on the basics of CRISPR/Cas9 and its application in sugarcane genome improvement. Last but not least, different potential limitations that might emerge during the deployment of this technique for genetic improvement of non-cultivated grass species have been objectively highlighted.

## 2 Non-cultivated grasses of economic significance

Grasslands (also known as prairie, savanna, steppe, pampas, etc.) are the areas dominated by grasses (Poaceae family) and different sedges of Cyperaceae family (Iqbal et al., 2022; Ijaz et al., 2023). Recently, grassland conservation has emerged as one of the biggest challenges due to their conversion into croplands (Wen et al., 2018; Yu et al., 2019). Previously, conservation efforts have generally aimed at preventing the loss of grass species, soil degradation, and fragmentation of grasslands for ensuring their long-term sustainability (Dong et al., 2019; Iqbal et al., 2022). Contrastingly, initiatives such as introduction of more efficient grass species and grazing systems along with implementation of sustainable land management techniques are needed for their conservation. In addition, grassland development in a broader economic perspective might involve initiatives to diversify and integrate varying sources or services through the promotion of tourism and affiliated industries (dairy, honey, and medicine) that are compatible with the conservation and sustainable use of grasslands (Saleh and Karwacki, 1996). Therefore, one of the biologically feasible ways of achieving grassland conservation and development could be genetic improvement of native grass species regarding which persistent research efforts are lacking so far. **Table 1** presents numerous non-cultivated grasses that hold bright economic perspectives (as biofuel, feed for ruminants, and beverages, and for medicinal use and aesthetic purposes); however, their productivity and nutritional value enhancement through gene editing is still awaited. Therefore, this study proposes to employ modern gene editing techniques of sugarcane especially CRISPR/Cas9 for genome editing of non-cultivated grasses.

**Table 1:** Different non-cultivated grass species, their prevalence regions, and prospective uses of economic significance as reported by Iqbal et al. (2022).

Grass species	Prevalence countries	Targeted traits for genetic improvement
Wild sugarcane/Kans grass (*Saccharum spontaneum*)	Panama, China, Pakistan, India, Nepal, Bhutan, and Fiji	Robust canopy development must be acquired under abiotic stresses through genetic manipulation as it is relished as a vegetable and can be used in house fencing and hut/roof thatching.
Little bluestem [*Schizachyrium scoparium* (Michx.) Nash]	North American countries	It might be improved to serve as an excellent biofuel crop having tolerance against heat and drought stress
Japanese sweet flag (*Acorus gramineus*)	United States of America and other North American countries	Aesthetic grass that needs genetic improvement for abiotic stresses (especially heat and drought) tolerance
Signal-grass (*Brachiaria racemose*)	Australia, India, Pakistan, China, South Africa, and many countries of Southern Europe	Nutritious feed for livestock especially higher protein content and digestibility along with lower fiber content
Switch-grass (*Panicun virgatum*)		Regeneration capacity and robust regrowth must be acquired through targeted genome editing
Lemon grass (*Cymbopogon citratus*)	India, Pakistan Philippines, China, Sri Lanka, Madagascar, Indonesia, United Kingdom, and many Central American countries	It might be genetically improved as an aromatic herb having brewing qualities, and is a source of essential oils and has medicinal uses including preparation of traditional antifungal, anti-bacterial, and antipyretic medicines
Sand bluestem (*Andropogon hallii* Hack.)	North American countries	Bioenergy grass; its drought tolerance needs to be acquired through genetic manipulation
Cogon grass (*Imperata cylindrica*)	United States of America, Argentina, and Peru	Ornamental grass; higher flowering potential needs to be achieved
Giant reed (*Arundo donax*)	Turkey, Israel, and Lebanon	Gene editing required to increase feasibility of its utilization of biofuel production
Pink muhly grass (*Muhlenbergia capillaris*)	Argentina, Chili, Peru, and United States of America	Environmental friendly (low input requiring) ornamental grass that needs genetic manipulation to increase flowering
Big bluestem (*Andropogon gerardii Vitman*)	The entire North American continent	Biofuel production
Chinese silver grass (*Miscanthus sinensis*)	China, USA, Brazil, and Canada	Ornamental grass; early flowering potential is highly desirable.
Eastern gamagrass (*Tripsacum dactyloides*)	North American countries like United States of America and Canada	Gene editing might convert it into a valuable raw material for bioenergy production
Bermuda grass (*Cynodon dactylon*)	China, India, Pakistan, Bangladesh, Brazil, and Chile	Forage (green succulent and preserved as hay or silage) for ruminants, whereas gene editing is needed to increase protein content and overall biomass production
Pycreus grass (*Pycreus flavidus*)	Pakistan, Iran, Turkey, China, Afghanistan, India, Israel, South Africa, Iraq, Lebanon, and Syria	Genetic improvement might increase biomass production and nutritional quality especially protein and ash content
Hairy crabgrass (*Digitaria sanguinalis*)	India, China, Pakistan, Brazil, and Argentina	Nutritious feed for dairy animals if gene editing effectively improves biomass production and regrowth potential
Miscanthus (*Miscanthus* sp.)	Turkey and other Mediterranean countries	Genetic improvement required to increase its utility as a biofuel grass
Job’s tears (*Coix lacryma-jobi*)	Southeast Asian countries like Philippines and Vietnam	Supplementary material for bakery products such as porridge and biscuits; medicinal uses for treating wounds, urinary tract infection, and blisters; it also has brewing quality
Prairie cordgrass (*Spartina pectinate*)	United States of America, Canada, and other North American countries	Bioenergy production

## 3 Sugarcane (a C4 grass) morpho-anatomical features and pertinence

Sugarcane (*Saccharum officinarum*) belongs to the genus *Saccharum* that entails many species such as *S. robustum*, *S. officinarum*, *S. barberi*, *S. edule*, *S. sinense*, and *S. spontaneum* (Tew and Cobill, 2008; Taparia et al., 2012; Hussin et al., 2022). These are genetically related to family Poaceae members such as sorghum, Miscanthus, and Erianthus (Saleh and Karwacki, 1996). It is a tropical and subtropical perennial C4 grass (Byrt et al., 2011) that is primarily grown for its high sugar content especially in China, Brazil, India, Thailand, Pakistan, and many other countries of Africa and Americas (Iqbal and Iqbal, 2014; Iqbal and Saleem, 2014; Iqbal et al., 2015). Sugarcane has been classified among the most productive cultivated grasses in modern input-intensive farming systems owing to its superior and unprecedented light, water, and nitrogen use efficiencies (Weeks, 2017). Apart from sugar, this perennial grass also finds its use in the production of ethanol, particularly in countries such as Brazil that promote biofuel production (Iqbal and Iqbal, 2014). Additionally, various by-products such as molasses are produced during sugar-making, which are used for producing ethanol, rum, etc (Iqbal and Saleem, 2014; Ko et al., 2018). Moreover, bagasse (the fibrous residue left after juice extraction) is another useful by-product of sugar production that is used for power and biofuel generation (Tew and Cobill, 2008; Mohan et al., 2020) along with serving as a raw material in paper and board production (Iqbal and Saleem, 2014; Eid et al., 2021).

**Figure 2:**
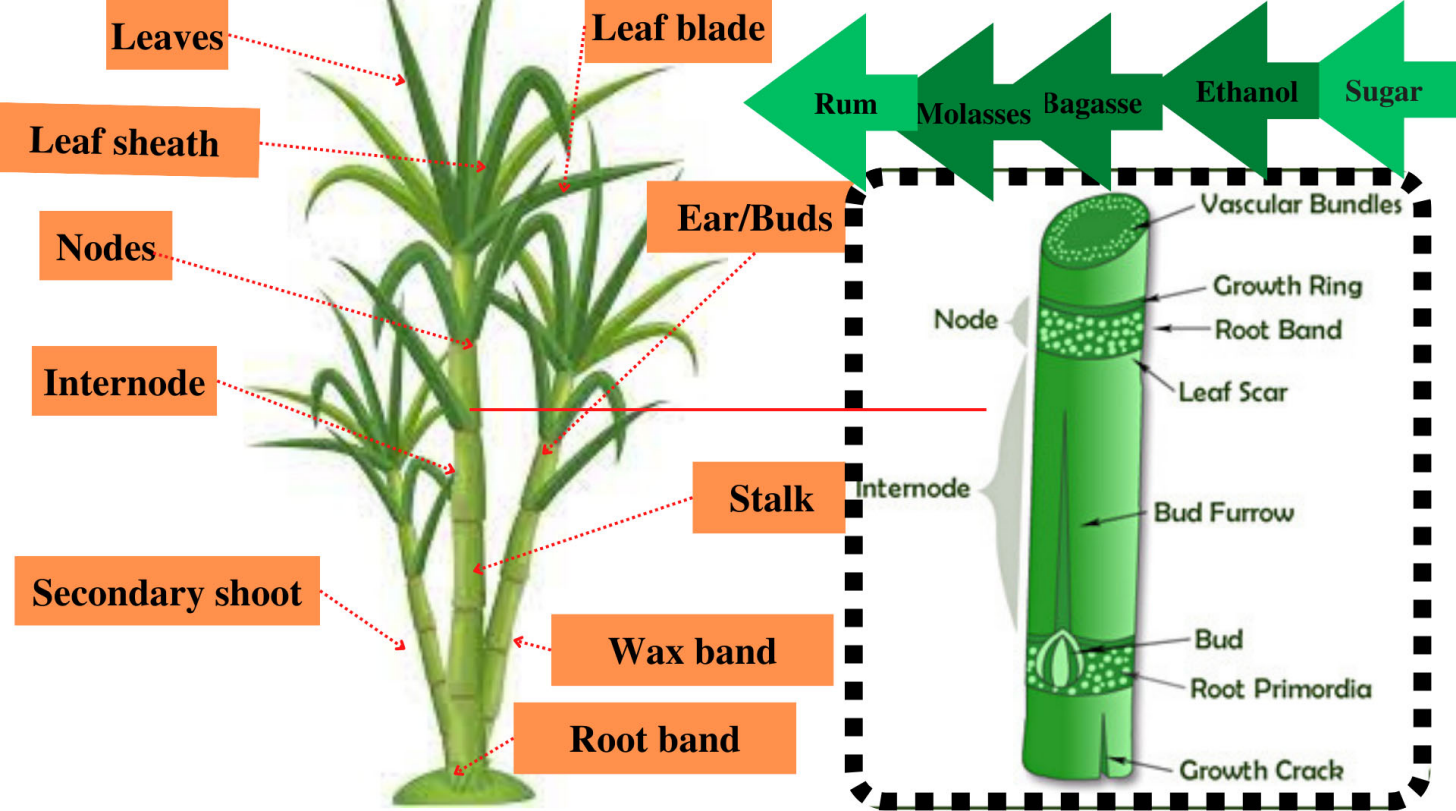
Prominent morphological features (leaf attributes including number of leaves, leaf sheath, and blade thickness along with number of nodes and inter-nodal distance) of the sugarcane plant and the anatomical features of cane/stalk (root primordia, which give rise to the root system, vascular tissues developing in xylem and phloem for transportation of water, nutrients and sugars in a source–sink relationship, leaf scar that serves as a prime feature for cultivar identification in the absence of leaves, etc.) that have been focused on in modern breeding and genetic improvement efforts along with different by-products (molasses, ethanol, bagasse, etc.) prepared directly from sugarcane.

The frequent occurrence of drought and other CCs have recently imposed pronounced deleterious effects on cane yield of elite cultivars (Andrade et al., 2014; Li et al., 2019). In addition, disruption of rainfall patterns and declining availability of irrigation water are slicing the yield of this higher water requiring cultivated grass (Trujillo et al., 2009; Begcy et al., 2012; Ferreira et al., 2012; Lin et al., 2014; Zhu et al., 2021). Persistent genome editing efforts have been made for improving the agro-botanical traits (enhanced number of leaves and leaf blade area for increasing the rate of photosynthesis, number of nodes and inter-nodal distance, cane diameter, and stronger network of root band to prevent lodging as portrayed in **Figure 2**) of sugarcane for imparting resilience against weather shifts and shortening of frost-free periods (Enriquez et al., 2000; Mohan, 2016; Nerkar et al., 2018). **Figure 2** illustrates prominent morphological and anatomical features of sugarcane plant that have remained the focus of modern breeding and genome editing efforts. The increase in number of nodes (distinct joints on which leaves, buds, and branches emerge) and intermodal distance, improved leaf sheath area, and the higher number of leaves, nodes, and buds per plant resulted in lesser disease attack and herbicide tolerance and in greater light, water, and nutrient absorption, conversion, and use efficiencies in sugarcane (Enríquez-Obregón et al., 1998; Gilbert et al., 2005; Tiwari et al., 2010; Gentile et al., 2015; Mohan, 2017; Oz et al., 2021). Likewise, genetic improvement of anatomical traits especially root primordia (embryonic structures that give rise to roots) and vascular bundles (complex tissues called xylem and phloem, which are the channels for transportation of water, nutrients, and sugars) tends to increase growth, yield attributes, and cane yield. Therefore, it is suggested that these improved traits of sugarcane hold bright perspectives to utilize genome editing techniques for boosting the morphological traits especially higher leaf area and plant height to promote photosynthesis efficiency for producing greater biomass, higher stem diameter, and extended root band to prevent lodging of non-cultivated grasses as well.

## 4 Gene editing tools and the CRISPR/CAS9 protocol for genetic improvement of major cultivated grasses

**Figure 3:**
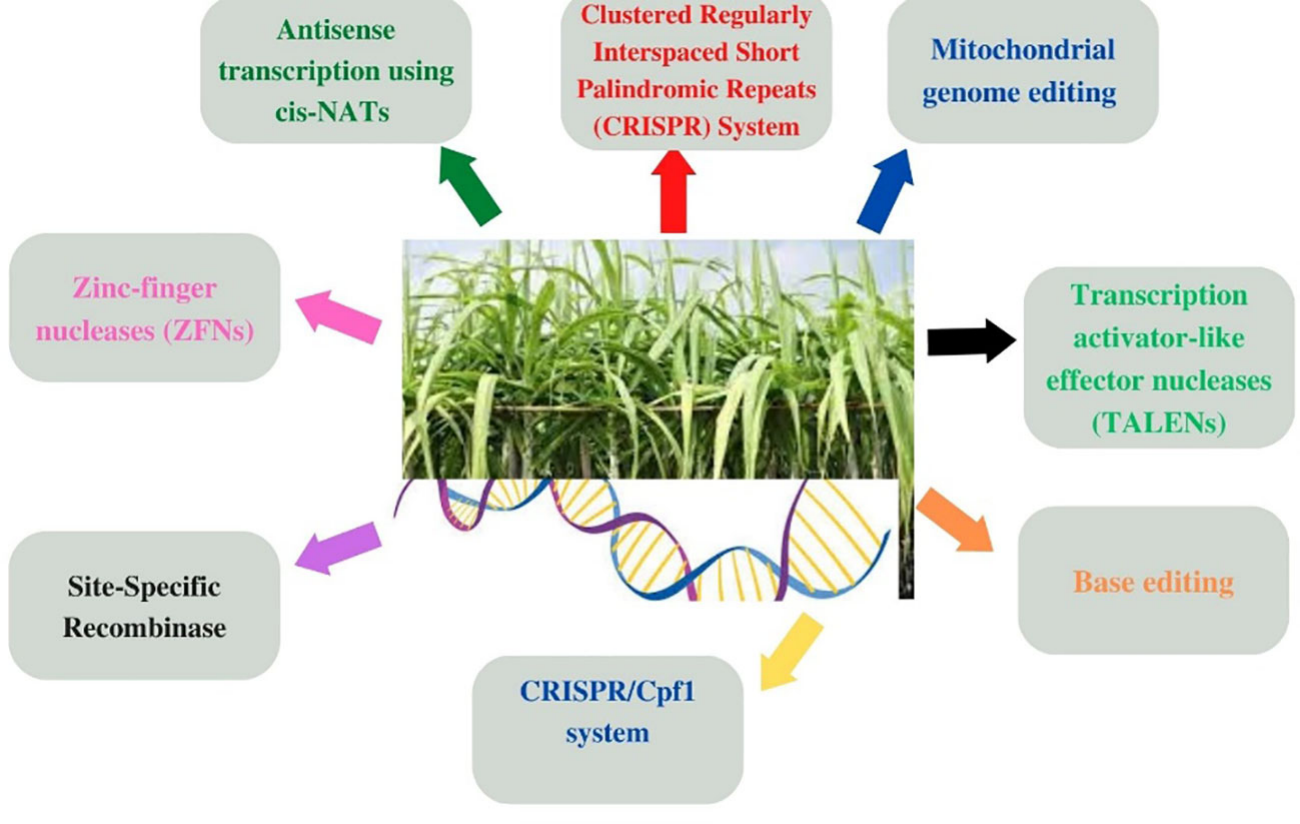
Genetic engineering approaches involving different gene editing techniques used for the genetic improvement of major cultivated grasses (wheat, maize, rice, sugarcane, barley, etc.) by acquiring desired morphological traits as depicted in **Figure 2** through gene insertion and/or knocking them out for inducing genetic manipulation and transformation.

Different gene editing tools such as mitochondrial genome editing, anti-sense transcription, and zinc-finger nuclease techniques have been previously employed to acquire the desired traits in sugarcane (**Figure 3**). Moreover, other genetic tools such as site-specific recombinase, base editing (Yin et al., 2015; Zong et al., 2017), and transcription activator-like effector nucleases (TALENs) have also been employed for gene’s insertion and/or knocking (Jung and Altpeter, 2016; Kannan et al., 2018) in order to acquire desired morphological traits and improve cane yield, sucrose recovery, etc., but these have demonstrated limited efficacy owing to off-targeting (Peng et al., 2015; Ma et al., 2016; Zaidi et al., 2017). This situation necessitated the development of more advanced genetic tools such as the CRISPR technique (Aitken and McNeil, 2010; Mao et al., 2013; Shan et al., 2018; Hussin et al., 2022) for the gene editing of sugarcane.

Originally, CRISPR/Cas9 was discovered in bacteria and archaea immune systems having a role in detecting and subsequently degrading the invasive DNA from bacteriophages and plasmids (Peng et al., 2015; Ma et al., 2016). Recently, CRISPR has been developed as a revolutionary gene editing technique that performs precise modification of DNA within the host’s genome (Hussin et al., 2022; Riaz et al., 2022; Krishna et al., 2023). The Cas9 part refers to the CRISPR associated protein 9, which serves as molecular scissors. It encompasses two regions, namely, the recognition (REC) lobe and the nuclease (NUC) lobe. Additionally, the REC lobe contains two multi-helix domains that are called REC1 and REC2, which are essential to bind with both guide RNA and target DNA (Xing et al., 2014; Ren et al., 2021). Moreover, REC1 contains α-helical structures of 25 α-helices and 2 β-sheets, while in contrast, REC2 is composed of six-helix structures and gets embedded within the REC1 domain. Similarly, NUC lobe entails three domains called RuvC, HNH, and PAM (protospacer adjacent motif) interacting domains. To cut the DNA’s double strands, firstly, REC lobe triggers sgRNA and DNA binding, whereas the RuvC and HNH domains facilitate to precisely cut target DNA’s complementary as well as non-complementary strands. Another vital component of the system is guide RNA, which is composed of two elements, namely, CRISPR RNA (crRNA) and trans-activating CRISPR RNA (tracrRNA) (Gasparis et al., 2018; Chen et al., 2019). Interestingly, the crRNA is an 18- to 20-base-pair-long sequence that recognizes, specifies, and ensures binding with the target DNA (Barrangou et al., 2007), whereas the tracrRNA (a twisted structure) tends to bind the scaffold for Cas9 nuclease. However, the tracrRNA sequence must be partially complementary with one of the crRNA segment (Ren et al., 2021).

**Figure 4:**
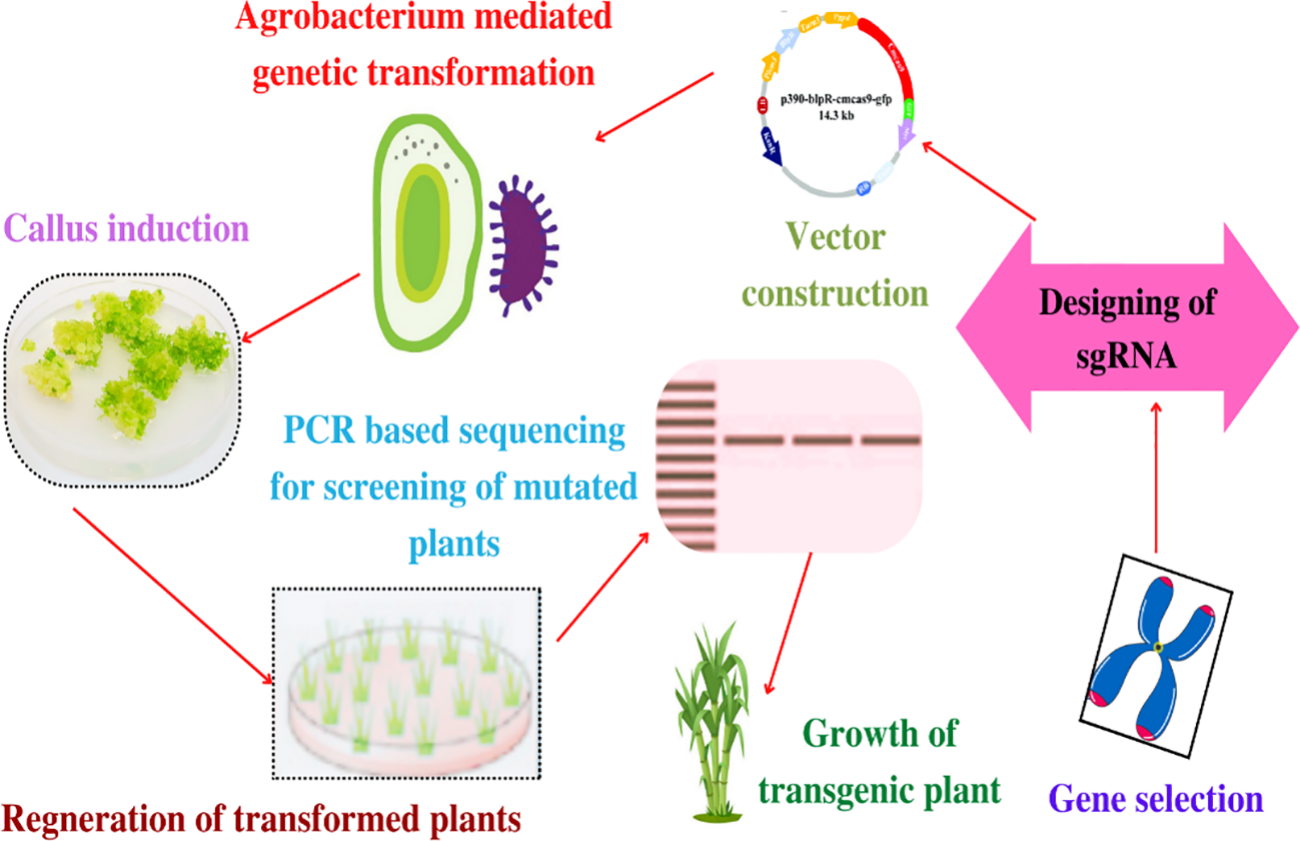
The schematic working protocol of the agrobacterium-mediated CRISPR/Cas9 technique (starting from gene selection and the design of guided RNA and terminating with the growth of transgenic plants after going through agrobacterium-mediated genetic transformation) for producing transgenic elite sugarcane cultivars having the desired agro-botanical and morphological traits.

There are different steps involved in the CRISPR/Cas9 working protocol (Mao et al., 2013; Osakabe et al., 2016). The first step involves designing a single guide RNA (sgRNA), which is a synthetic RNA molecule that is compatible with the target DNA sequence. The sgRNA has a vital function as it locates the specific gene or region of interest within the genome of the host organism. The next step is target recognition, whereby sgRNA gets associated with the Cas9 protein (Xing et al., 2014). The resulting complex serves as a pair of molecular scissors that gets triggered for searching the target DNA sequence within the genome of target host. Thereafter, DNA cleavage occurs by the Cas9 protein that induces a break in the DNA at the precise location (Svitashev et al., 2015; Galli et al., 2022). This DNA cleavage tends to trigger the natural repair mechanisms within the cell, which attempts to repair the break with the help of either homology directed repair (HDR) or non-homologous end joining (NHEJ). Interestingly, the NHEJ holds potential to introduce small insertions or deletions, which leads to gene disruption (Ren et al., 2021). In contrast, the HDR provided with a repair template might allow the introduction of specific genetic modifications (Xing et al., 2014; Tang et al., 2017; Miao et al., 2018). For introducing breaks in the double strands, CRISPR needs PAM sequence in the target DNA adjacent to the protospacer complementary sequence, which is a short sequence (2–6 bp) and precedes by the sequence of targeted DNA. This constitutes a serious limitation in its design and has raised the need to develop variants of CRISPR tools having alternative PAM requisites (Xing et al., 2014; Lawrenson et al., 2021). Interestingly, the Cas9 nuclease from the type II CRISPR/Cas9 system of *Streptococcus pyogenes* is the most frequently used system that requires PAM sequence for DNA targeting and an NGG (N, any nucleotide; G, guanine) component (Jinek et al., 2012). **Figure 4** illustrates the schematic working protocol of the CRISPR/Cas9 technique (starting from gene selection and designing of guided RNA and terminates with the growth of transgenic plants) for gene editing of sugarcane. Interestingly, the working efficacy of gene editing depends on two prime components involved in a typically engineered CRISPR/Cas9 system, a Cas (which is an endonuclease protein) and an sgRNA (that is basically a 20-nucleotide sequence) for guiding the Cas enzyme toward the target sequence in order to introduce double-stranded break (DSB) (Xing et al., 2014; Vlcko and Ohnoutkova, 2020).

The components of the CRISPR genetic system could be delivered into the target plant’s genome in the format of DNA, mRNA (*in vitro* transcripts or IVT), and proteins (Eid et al., 2021). The delivery techniques for CRISPR components include Agrobacterium-mediated infection, agro-infiltration, biolistics (also known as particle bombardment), electroporation, virus-mediated transformation, and PEG-based transformation, which is also referred to as protoplast-based transformation (Liang et al., 2014; Lin et al., 2014, Yin et al., 2015; Malnoy et al., 2016; Zaidi et al., 2017; Bhowmik et al., 2018). The RNP complex direct delivery has been reported to eliminate the risk of foreign DNA introduction into the genome of the host plants (Wolter and Puchta, 2017; 2018). Interestingly, pre-assembled RNP (Cas9-Grna) delivery was precisely attempted in cells (Cho et al., 2013). Later on, Cas9-gRNA RNPs have been successfully delivered into protoplasts by using the PEG-mediated delivery system that was derived from somatic tissues of tobacco, rice, petunia, grapevine, lettuce, apple, and potato Malnoy et al., 2016; Weeks, 2017). Recently, by using the biolistic bombardment protocol, Cas9-gRNA RNPs have also been delivered into maize and wheat embryo cells (Svitashev et al., 2015; Liang et al., 2016).

There are numerous generalized applications of the CRISPR/Cas9 technique such as gene editing with precise modification of specific genes (addition, deletion, or replacement of DNA sequences) (Westra et al., 2013; Manghwar et al., 2019; Milner et al., 2020). Disease modeling has emerged as another vital application of the CRISPR/Cas9 technique through the creation of model organisms with specific genetic mutations in order to diagnose the potential causes and develop feasible treatments (Kumar et al., 2018; Kim et al., 2022). Another interesting application of this technique is to study gene functions through selective disruption of genes and thereafter observing the resulting effects of altered genes. It is being used to develop genetically modified organisms (GMOs) having desired traits such as pest resistance (Kim et al., 2022). **Table 2** illustrates different applications of this technique for the genetic improvement of cultivated grasses (wheat, rice, maize, barley, and sorghum). The increment in yield and quality of different cultivated grasses (wheat, maize, and sorghum) and imparting resistance against biotic and abiotic stresses have been achieved by employing this novel technique (Azevedo et al., 2011; Svitashev et al., 2015; Shimatani et al., 2017; Zong et al., 2017; Bhowmik et al., 2018; Holubová et al., 2018; Li et al., 2019). Such genetic improvements might be attained in non-cultivated grasses as well; however, these might not give desired results for non-cultivated grasses having intricate genetic makeup. Over time, multiple variants of Cas9 and gRNA have been developed (Nishimasu et al., 2018; Chen et al., 2019; Walton et al., 2020), which could hold bright perspectives in genome editing of non-cultivated grasses.

**Table 2:** Major cultivated grass improvement (yield and quality enhancement along with imparting tolerance against biotic and biotic stresses) using the CRISPR/Cas9 gene editing technique.

Crops	Technical name	Genes involved	Relevant functions	Reference
Yield improvement				
Maize	*Zea mays*	Wx1	Yield enhancement	Waltz, 2016
Maize	*Zea mays*	LIG, MS26, MS45	Induced male part sterility in maize that prevents fertilization and ultimately no cob development occurs	Svitashev et al. (2015)
Barley	*Hordeum vulgare*	HcCKK1	Associated with boosting the number of grains per spike of barley crop	Holubová et al. (2018)
Barley	*Hordeum vulgare*	HvCKX1	Enhanced the grain yield by converting hulled grains into naked grains, which led to higher grain weight and number per plant of barley	Gasparis et al. (2018)
Quality enhancement				
Wheat	*Triticum aestivum*	Alpha-gliadin	Regulates the biosynthesis of gluten protein in wheat grain	Brandt et al. (2017); Bhowmik et al. (2018)
Barley	*Hordeum vulgare*	GST and IPI	Associated with accumulation recombinant proteins in barley grains	Panting et al. (2021)
Barley	*Hordeum vulgare*	HvCKX1	Tend to improve brewing quality of grains	Gasparis et al. (2018)
Maize	*Zea mays*	ZmIPK	Involved in the biosynthesis of phytic acid content	Liang et al. (2014)
Sorghum	*Sorghum bicolor*	Alpha-kafirin	Assist to improve the biosynthesis and digestibility of lysine	Li et al. (2018a)
Rice	*Oryza sativa*	SBEIIb	Boosts the biosynthesis of amylose content	Sun et al. (2017)
Tolerance against biotic stresses				
Wheat	*Triticum aestivum*	TaABCC6 ABC	Associated with imparting resistance against *Fusarium* head blight	Bhowmik et al. (2018)
Wheat	*Triticum aestivum*	TaNFXL1	Enables plant to resist the attack of diseases like *Fusarium graminearum*	Brauer et al. (2020)
Rice	*Oryza sativa*	OsSWEET11	Associated with developing resistance in rice seedling against a wide range of plant pathogens	Xing et al. (2014)
Rice	*Oryza sativa*	OsWRKY93 and OsMORE1a	Involved in imparting resistance against viral and fungal diseases especially tungro disease	Kim et al. (2022)
Rice	*Oryza sativa*	eif4g	Offers resistance in rice seedlings against viral tungro disease	Macovei et al. (2018)
Maize	*Zea mays*	ALS	Imparts resistance against broad-spectrum herbicides	Svitashev et al. (2015)
Barley	*Hordeum vulgare*	HvMORC1	Makes barley plants resistant to the invasion of *Fusarium graminearum*	Galli et al. (2022)
Barley	*Hordeum vulgare*	HvMORC6a	Tend to impart resistance against oomycetes	Galli et al. (2022)
Tolerance against abiotic stresses				
Rice	*Oryza sativa*	OsMYB1	Enables rice plants to survive in the wake of abiotic stresses (heat, drought, chilling, salinity, heavy metal toxicity, water logging, etc.)	Mao et al. (2013)
Rice	*Oryza sativa*	OsARM1 and OsNramp5	Imparts resistance against heavy metal (especially cadmium and arsenic) toxicity	Tang et al. (2017)
Rice	*Oryza sativa*	OsPYL	Modulates tolerance level against heat stress	Miao et al. (2018)
Barley	*Hordeum vulgare*	Inositol-kinase kinase tetrakisphosphate 1-	Imparts tolerance against stress caused by salinity in salt-affected soils (saline, sodic, and saline-sodic soils)	Vlcko and Ohnoutkova (2020)
Barley	*Hordeum vulgare*	HvPM19	Regulates the dormancy of barley grains under stressful conditions (heat, drought, water logging, salinity, etc.)	Lawrenson et al. (2021)

## 5 CRISPR (Cas9, Cas12a, Cas12b, and SpRY) variants

In the CRISPR gene editing system, the guide RNA’s protospacer motif tends to provide target specificity (Liang et al., 2014; Gasparis et al., 2018). However, compatible PAM sequence is a pre-requisite to trigger the cleavage of the targeted DNA region. Additionally, a GC-enriched site is required by PAM prototypical Cas9 derived from *S. pyogenes* (SpCas9), which reduces flexibility targeting. The PAM presence restrains potential site access, which results in off-targeting. Notwithstanding, Cas enzymes hold potential for target site recognition, which increases the flexibility of target sites (Nishimasu et al., 2018; Miller et al., 2011). Recently, numerous variants of endonuclease enzyme have been developed including Cas12a and Cas12b (Chen et al., 2019; Ming et al., 2020). However, akin to Cas9, these variants are not without PAM requirement and rely on PAM’s T enriched at the 5′-end in the form of TTTV. Recently, Walton et al. (2020) have reported overcoming this limitation through the development of the SpCas9 enzyme variant, which is a structure-guided engineered variant and referred to as SpRY. This newly developed variant holds potential to target the genomic DNA without requiring PAM and might be declared as nearly PAM-less variant. Thereafter, Ren et al. (2021) have reported that SpRY remained equally effective in rice by successfully targeting a large number of NNN PAM sites (NAN/NGN/NCN/NTN). Contrastingly, it was observed that Cas9 was unable to edit a number of relaxed PAM sites and was pronouncedly less efficient in comparison to SpRY for non-canonical PAM sites. Moreover, it was reported that SpRY induced larger deletions (five base pairs at relaxed PAM sites), which was impossible to achieve by using the Cas9 gene editing tool. Interestingly, the PAM requirement elimination induced self-editing in CRISPR-Cas T-DNA, which led to either inactivation or modification of sgRNA (Zong et al., 2017; Miller et al., 2011; Milner et al., 2020).

Likewise, the CRISPR-mediated genome editing tool for single base editing has also been applied in a variety of cultivated grasses. For instance, adenine and cytosine base editing has been effectively optimized in cultivated grasses like rice, wheat, and maize for base editing (Shimatani et al., 2017; Zong et al., 2017; Li et al., 2018). However, those were found inefficient owing to off-targeting effects while more research is needed to enhance the efficiency of base editing tools in monocots. Recently, in rice, SpRY-PmCDA1 (PAM-less C-to-T nucleotide editor) remained effective in converting a C-to-T base (Ren et al., 2021). Thus, it has been inferred that CRISPR-associated SpRY enzyme’s expanded target range might be further harnessed for base editing (nucleotide-level) with high accuracy. This can be achieved by using cytosine base editors at the relaxed PAM (first to sixth base of protospacer) of the SpRY. It was impossible to achieve this using the traditional C-to-T base editors owing to the peculiar distance requirement of editing windows (Manghwar et al., 2019). In contrast, the SpRY-based adenine base editor has demonstrated higher efficiency for A-to-G conversion by using fourth to eighth bases of the protospacer in the editing window (Ren et al., 2021). Hence, it might be inferred that by using SpRY-based editors, a comparatively hefty number of options regarding base edits have become available now. It is worth mentioning that in the CRISPR-based system, PAM tends to differentiate specific Cas enzyme non-self DNA sequences (Westra et al., 2013). The CRISPR tool having PAM-less targeting capacity could limit and restrict self-editing, which could be utilized for secondary off-targeting. The off-target in transgenic rice lines could be prevented by a self-targeting gRNA vector (Ren et al., 2021). These shortcomings compel further investigations pertaining to structural engineering for application in different systems such as single base editing using SpRYABEs (Walton et al., 2020).

## 6 CRISPR/Cas9 in sugarcane and potential application for non-cultivated grass improvement

The genome size of sugarcane has been estimated to be over 10 Gbp, wherein genes exist in 10–12 allelic forms. Interestingly, depending on a specific cultivar’s ploidy level, monoploid genome size has been estimated to be approximately 800–900 Mb (Zhang et al., 2012; de Setta et al., 2014; Hussin et al., 2022). Because of its high polyploidy (*x* = 10–13; 2*n* = 100–130), interspecific, heterozygous, and aneuploidy nature, the genome of sugarcane tends to decelerate the gene editing attempts intended for crop improvement (Le Cunff et al., 2008; de Setta et al., 2014; Oz et al., 2021). Moreover, modern elite cultivars of sugarcane exhibit high level of polyploidy and heterozygosity that necessitate the vegetative propagation of sugarcane in order to prevent allele loss and inhibit detrimental allele accumulation during the process of meiosis (Ali et al., 2019; Krishna et al., 2023). However, most of sugarcane’s parental clones lacking pollen fertility and flowering synchrony have been improved using genetic engineering approaches (Hoang et al., 2015). There have been continuous research efforts to genetically improve sugarcane for boosting cane yield and sucrose recovery (Tew and Cobill, 2008; Hamerli and Birch, 2011; Krishna et al., 2023).

**Figure 5:**
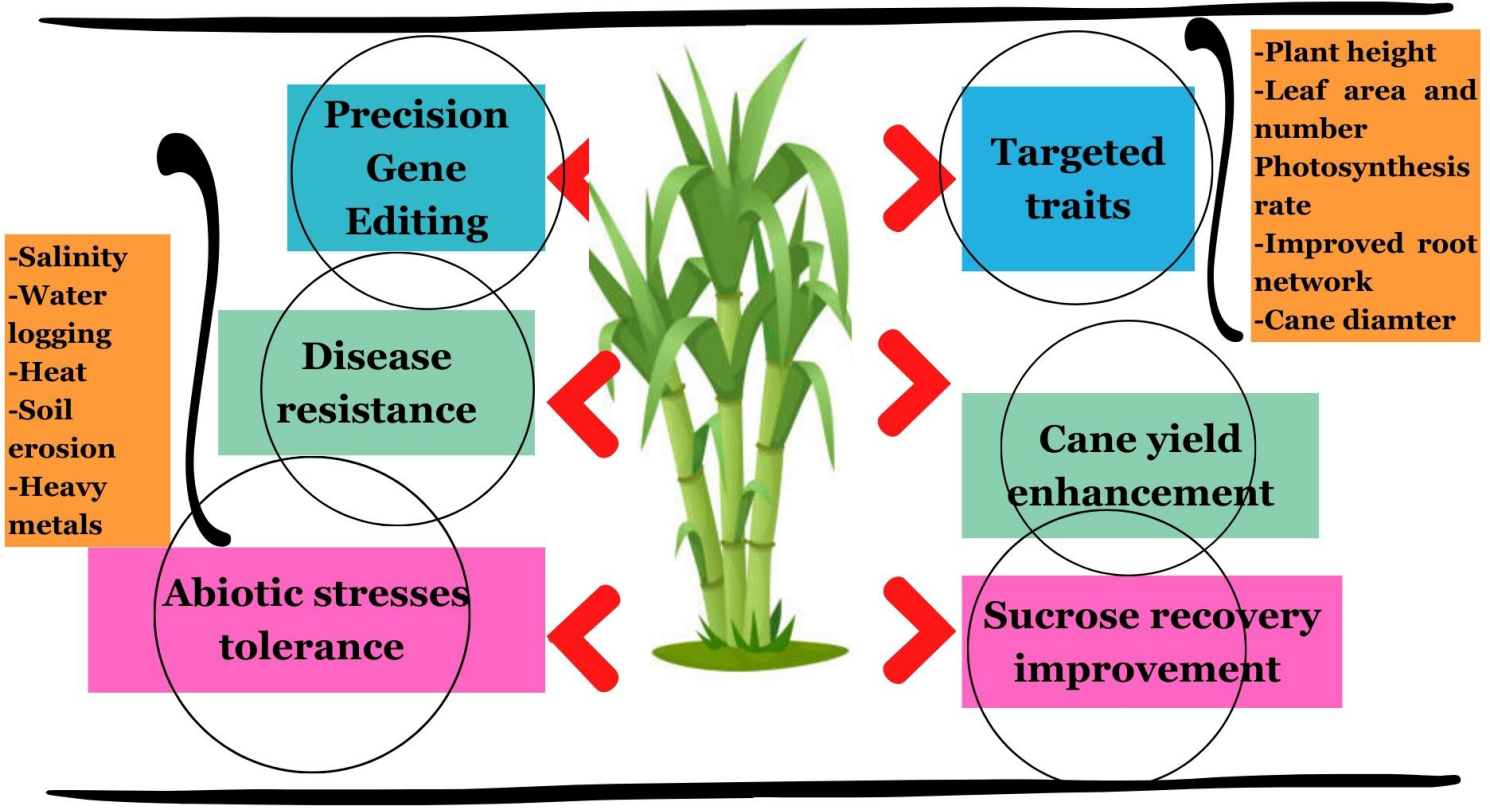
Prominent applications of CRISPR/Cas9 in sugarcane for precise gene editing to acquire the desired traits, especially improvement of morphological attributes (plant height, leaf number per plant, leaf area, cane diameter, etc.), cane yield, sucrose recovery percentage, and tolerance against abiotic stresses (salinity, heat, water logging, soil erosion, and heavy metal toxicity).

Among major cultivated grasses, taking highly polyploidy sugarcane as a model crop might be a rational approach for genome editing of non-cultivated grasses due to the absence of mutagenesis in diploid grasses (**Table 2**). The functional redundancy in sugarcane is caused by homeologs and homologs that are present in a large number and restricted genome editing (Taparia et al., 2012; Weeks, 2017; Eid et al., 2021; Oz et al., 2021). However, co-mutated allele numbers are similar to RNAi, offering an unprecedented opportunity to produce a wide range of phenotypes (Eid and Mahfouz, 2016). The CRISPR variants have revolutionized the gene editing process and are being applied in various polyploidy crops including sugarcane for introducing precise genetic modifications with ultimate aims to improve yield, sucrose recovery, biofuel production, disease resistance, and abiotic stress tolerance (Azevedo et al., 2011; Taparia et al., 2012; Hoang et al., 2015; Ali et al., 2019). **Figure 5** presents some prominent applications of CRISPR/Cas9 in sugarcane for precision gene editing to acquire the desired morpho-physiological traits. Thus, this technique holds bright perspectives to increase the biomass yield, nutritional quality, and tolerance against biotic and abiotic stresses in non-cultivated grass species through precise screening and targeting of desired genes for acquiring the desired traits.

Among the specific applications of CRISPR/Cas9 in sugarcane, precise gene modification ranks top by using gRNA to target a specific DNA sequence and the Cas9 enzyme to cut the DNA (Eid and Mahfouz, 2016). In this way, it effectively helps acquire the desired traits such as increased sugar content and resistance against diseases (smut, rust, rot, etc.) (Hoy, 1994; Comstock, 2002; Fitch et al., 2001; Viswanathan and Rao, 2011; Hussain et al., 2018) and abiotic stresses (especially drought, heat, salinity, water logging, heavy metal toxicity, etc.) in an environmentally friendly manner (Gomathi et al., 2015; Tiwari and Lata, 2018; Baig et al., 2020; Rehman et al., 2021; Riyazuddin et al., 2022). Most importantly, this technique in sugarcane has been utilized for developing high-yielding cultivars that require fewer inputs including water, fertilizers, pesticides, etc. that might contribute to impart sustainability to modern intensive sugarcane farming systems. The CRISPR/Cas9 technique deployed in sugarcane as a part of broader efforts in agricultural biotechnology might prove vital in addressing the global challenges of food security, environmental sustainability, and crop resilience (Patade et al., 2008; Sengar et al., 2013; Meena et al., 2020).

Recently, Eid et al. (2021) inferred that the CRISPR/Cas9 technique remained effective in producing a rapidly scorable phenotype in highly polyploid sugarcane through multiallelic, targeted mutagenesis of magnesium chelatase. Likewise, this technique performed precise genome modifications in many elite varieties through bypassing the adverse meiosis in sugarcane (Weeks, 2017) and the same could be repeated in non-cultivated grasses of economic pertinence such as wild sugarcane, Bermuda grass, and Chinese silver grass. Likewise, Oz et al. (2021) reported that efficient and reproducible gene targeting in sugarcane was possible by enabling precise co-editing of multiple alleles via template-mediated and homology-directed repair of DNA double-strand breaks induced by the programmable nuclease CRISPR/Cas9 technique. Ultimately, the co-editing of three acetolactate synthase alleles that could confer herbicide tolerance was confirmed by Sanger sequencing through PCR amplicons. Thus, the CRISPR/Cas9 technique holds potential to precisely target non-cultivated grass species genome for creating tolerance against broad-spectrum herbicides especially in grassland areas adjacent to cultivated lands. It was also inferred that through the comparison of different quantities, delivery of the repair template suggested that exogenously supplied DNA’s excessive quantities might adversely impact the repair process in sugarcane. In addition, Azevedo et al. (2011) opined that CC has asserted extreme pressure on high water-demanding crops like sugarcane, while drought and heat stresses tend to reduce cane yield and sucrose recovery, while CRISPR/Cas9 might be utilized to impart tolerance against terminal heat stress and drought (Meena et al., 2020). This technique could be employed to precisely target the genome of non-cultivated grasses for improving their tolerance against heat and drought stresses. Patade et al. (2008) reported that drought and heat stresses result in salinity owing to higher volatilization from soil surface that causes salt accumulation and, resultantly, sugarcane growth; cane yield and sucrose content were significantly decreased. However, the CRISPR/Cas9 tool holds immense potential to produce elite genotypes of sugarcane having the potential to thrive well on salt-affected soils through precise mutagenesis in sugarcane (Sengar et al., 2013). Soil salinity tolerance in non-cultivated grasses might revolutionize the grassland conservation and development initiatives, leading to ensuring food security and poverty alleviation on a wide scale. Similar results have been reported for sugarcane gene editing for imparting tolerance against other abiotic stresses including water-logging, cold or chilling stress, and heavy metal toxicity using a precise genome editing technique like CRISR/Cas9 (Tiwari et al., 2010; Rehman et al., 2021; Riyazuddin et al., 2022).

Besides abiotic stresses, CRSISPR/Cas9 holds bright perspectives in producing elite genotypes of sugarcane having immense tolerance against biotic stresses. Numerous biotic stresses including weeds, diseases, and a wide range of insects have posed a serious challenge to sugarcane production as per their varietal potential (Hussain et al., 2018). Viswanathan and Rao (2011) reported the precise application of this technique for imparting tolerance against the fungal diseases of sugarcane such as wilt (the causative agent is *Fusarium sacchari*) and smut (caused by *Sporisorium scitamineum*) and red rot caused by *Colletotrichum falcatum*. The same goes for bacterial diseases including ratoon stunting and leaf scald along with sugarcane yellow leaf virus, which cause significant losses in sugarcane (Hoy, 1994; Fitch et al., 2001; Comstock, 2002). **Table 3** indicates different candidate genes identified through the CRISPR/Cas9 technique to impart tolerance against biotic and abiotic stresses. These successes might be utilized to initiate genetic improvement of non-cultivated grasses for imparting tolerance against viral, bacterial, and fungal diseases.

**Table 3:** Different candidate genes identified for imparting tolerance against rust and smut diseases along with abiotic stresses (drought, salinity, cold, or chilling stress and oxidative stress) in sugarcane through precise genome editing using the CRISPR/Cas9 technique.

Stress type	Candidate genes	References
Biotic stresses		
Rust caused by *Puccinia melanocephala* Syd.	Bru1	Asnaghi et al. (2004)
Smut caused by *Sporisorium scitamineum*	ScCAT1	Wu et al. (2023)
Abiotic stresses		
Salinity stress	miRNAs	Ferreira et al. (2012)
Salinity stress	ShPHT	Murugan et al. (2022)
Salinity stress	SodERF3	Trujillo et al. (2009)
Salinity stress	SoMYB18	Shingote et al. (2015)
Chilling/Cold stress	SspNIP2	Park et al. (2015)
Chilling/Cold stress	ShGPCR1	Ramasamy et al. (2021)
Drought stress	ScLoX	Trujillo et al. (2009); Andrade et al. (2014); Lin et al. (2014)
Drought stress	SoP5CS	Li et al. (2018)
Drought stress	SoACLA-1	Zhu et al. (2021)
Drought stress	miRNAs	Ferreira et al. (2012)
Oxidative stress	Scdr1	Begcy et al. (2012)
Oxidative stress	ScAPX6	Liu et al. (2018)
Oxidative stress	ScDir	Liu et al. (2018)

## 7 Limitation of CRISPR/Cas9 for non-cultivated grass improvement and future perspectives

Recently, it has become evident that CRISPR/Cas9 has offered unique efficiency with unmatched precision in gene editing of polyploidy crops like sugarcane (Bhowmik et al., 2018; Hussin et al., 2022); however, its application for non-cultivated grasses’ genetic improvement might raise few technical, ethical, and safety concerns. The key technical limitations of CRISPR/Cas9 might include challenges like achieving 100% precision in gene editing (Ali et al., 2015; Zhang et al., 2019), because few cells could avoid desired genetic modifications leading to low precision in non-cultivated grass species. This could become a serious limitation in cases where high accuracy is crucial and highly desired and the same could be a serious challenge in case of grass mutagenesis. Another limitation might be off-target effects as the Cas9 protein could bind and cleave the target DNA at unintended locations (Wang et al., 2014; Baltes et al., 2015), leading to undesired genetic changes, which, in turn, lead to potentially harmful consequences in terms of biomass production and nutritional value of grass species. For gene editing in polyploidy crops including tetraploid cotton (*Gossypium hirsutum*), hexaploid wheat (*Triticum aestivum*), and sugarcane, mutations generally occur in homoeoallele subsets targeted by the same sgRNA (Wang et al., 2017; Zhang et al., 2019). Furthermore, polyploidy due to Mendelian genetics makes transmission and stacking of first-generation mutations harder and even impossible. In addition, one of the prime limitation of the CRISPR/Cas9 technique could be CRISPR/Cas9 component delivery (Hussin et al., 2022) into the target genome of grass species, which would seriously compromise the efficacy and accuracy of the whole gene editing process, while serious research efforts could be required to optimize the delivery system of CRISPR for polyploidy grasses. Likewise, delivery of CRISPR/Cas9 components selectively to specific cell types within a complex genome of host grasses might remain a daunting challenge as that of sugarcane.

Additionally, insertion of large DNA sequences continues to remain one of the pronounced challenges (Liang et al., 2016; Gao et al., 2020; Eid et al., 2021; Oz et al., 2021), which decreases its ability to add large regulatory elements into the targeted genome of non-cultivated grasses. Moreover, one of the limitations of the CRISPR/Cas9 technique is mosaicism (Yin et al., 2015) whereby this technique could induce non-uniform genetic modification in different cells of non-cultivated grasses; thus, before employing this technique for grass species, optimization of the CRISPR system might be required. Although not in sugarcane, but the immune system of many hosts has responded negatively to CRISPR/Cas9 components (Baltes et al., 2015), limiting the efficacy of the gene editing process, and the same could be happen in the case of a few non-cultivated grass species. Presently, gene editing by using CRSPR/Cas9 has assisted cultivated grass improvement by facilitating precise knock-in, triggering accurate knockout and desired replacement, and promoting planned point mutations and gene fine-tuning (Svitashev et al., 2015; Tang et al., 2017; Li et al., 2018; Miao et al., 2018; Gao et al., 2020). For non-cultivated grass improvement, the potential development of the CRISPR method to facilitate on-target editing and circumvent the vector self-editing that reduces off-targeting may be further explored. Although Cas9 is incapable to act in a PAM-less editing mode, its accuracy and efficiency have remained far better than SpRY, which highlights Cas9’s unterminated pertinence for genetic modification of grass species (Ren et al., 2021). Recently, SpRY has been developed as a more precise choice for exploring the genome of crop plants especially its application in rice as proved by its efficacy in terms of unconstrained targeting using PAM-less editing (Tang et al., 2017; Macovei et al., 2018). The application of Cas9 and SpRY is bound to inspire numerous exciting investigations including *in vivo* directed evolution for acquiring desired characteristics that bolster plant establishment against biotic and abiotic stresses under changing climate scenario.

Furthermore, there remain few ethical and social concerns regarding the potential application of the CRISPR/Cas9 technique in creating GMO crops (Malnoy et al., 2016; Zaidi et al., 2017). Previously, CRISPR/Cas9 use in sugarcane has also raised ethical and regulatory considerations (Ostengo et al., 2022), and the same could be expected for non-cultivated grass species as well. Different countries may not have regulations regarding the genetically modified grass species, which could delay the initiation and execution of genome editing programs. Furthermore, application of this technique could prompt the need to devise robust regulatory frameworks to ensure its responsible use and to avoid the potential unintended consequences of genome editing non-cultivated grasses. Despite unprecedented opportunities offered by the CRISPR/Cas9 technique regarding the precise genome editing, these limitations must be given due consideration before considering this technique for genetic improvement of non-cultivated grasses.

## 8 Conclusions

Owing to CC, global warming, rapidly increasing human population, and decreasing agricultural land area, it is about time to initiate out-of-the-box conservation strategies for grasslands. This goal could be effectively achieved through genetic improvement of native grass species in order to diversify and multiply their ecosystem services. Gene editing techniques might be utilized to genetically improve native grasses based on the pattern of cultivated grasses like sugarcane. Among the recent genetic techniques employed in sugarcane, CRISPR/Cas9 has emerged with an immense potential to precisely modify the specific genes in the target host’s genome with unprecedented accuracy and efficiency. This technique has produced marvelous results in sugarcane gene editing for acquiring desired traits like higher cane yield, sucrose recovery, and tolerance against biotic and abiotic stresses, and the same might be utilized for grass species of grasslands. Future research must strive to attain abiotic stress tolerance in non-cultivated grass species using the CRISPR/Cas9 technique and other desired characteristics including higher biomass productivity, regrowth capacity, nutritional quality (especially higher protein and digestibility and lower fiber content) of grasses for consumption as forage for ruminants, biofuel production potential, and flowering capacity. After genetic improvement, one of the vital aspects would be the introduction of new seeds into the grasslands that can be economically achieved through over-seeding. However, unlike cultivated grasses, future genome editing research has to face novel challenges like gene delivery issue, off-targeting, and limited efficacy of gene editing procedures, but such research struggles are bound to open new frontiers of genome editing of non-cultivated grasses, which might contribute to ensuring food security in the future.

## Author contributions

CL: Conceptualization, Writing – original draft, Writing – review & editing, Methodology, Project administration. MAI: Conceptualization, Writing – original draft, Writing – review & editing, Investigation, Resources.
